# Impact of Rectal Spacer on Toxicity Reduction in Men Treated With Proton Versus Photon Therapy^1^

**DOI:** 10.1016/j.ijpt.2024.100111

**Published:** 2024-06-20

**Authors:** Vishal R. Dhere, Subir Goyal, Jun Zhou, Nikhil T. Sebastian, Ashish B. Patel, Sheela Hanasoge, Pretesh R. Patel, Joseph Shelton, Karen D. Godette, Bruce W. Hershatter, Ashesh B. Jani, Sagar A. Patel

**Affiliations:** 1 Department of Radiation Oncology, Winship Cancer Institute, Emory University, Atlanta, Georgia, USA; 2 Department of Biostatistics and Bioinformatics, Emory University, Atlanta, Georgia, USA

**Keywords:** Rectal spacer, Rectal toxicity, Prostate radiation, Proton versus photon, Late toxicity

## Abstract

**Purpose:**

Rectal toxicity after prostate cancer (PCa) radiation therapy (RT) may be greater with protons compared with photon intensity-modulated RT, perhaps due to lateral penumbra and end-of-range uncertainty. Rectal spacers (RSs) have been shown to mitigate RT-associated acute/late rectal toxicity in men treated with photons. The relative benefit of RS in men treated with protons versus photons is unknown. We hypothesize that RS will confer greater bowel toxicity benefits in PCa treated with protons versus photons.

**Materials and Methods:**

We conducted a single institution, retrospective review of men receiving photon intensity-modulated RT or pencil-beam scanning proton RT for localized PCa. Four cohorts were compared: photon with or without RS, and proton with or without RS. Acute (<3 months), late (≥3 months), and most recent toxicity were compared among the 4 cohorts. The cumulative incidence of physician-reported grade 1 to 2 gastrointestinal (GI) toxicity (common terminology criteria for adverse events V5.0) was compared using χ^2^ or Fisher exact test. Patient-reported toxicity was evaluated using the International Prostate Expanded Prostate Composite Index-Clinical Practice and compared using linear mixed modeling.

**Results:**

In total, 164 patients were eligible for analysis: 38 photons without RS, 50 photons with RS, 26 protons without RS, and 50 protons with RS. The median follow-up was 17.6 months. In proton patients, acute (6.12% vs 30.77%, *P* = .009) and most recent (4.26% vs 26.09%, *P* = .01) G1-2 GI toxicity was lower with versus without RS. In photon patients, there were no significant differences in toxicity with versus without RS. No significant differences in patient-reported outcomes were observed with versus without RS in photon or proton groups.

**Conclusion:**

The rectal spacer was associated with lower G1-2 acute and most recent GI toxicity in men treated with protons; this difference was not observed in men treated with photons. While this study is limited by sample size, a relatively greater benefit of RS with proton versus photon therapy was observed.

## Introduction

Dose-escalated radiation therapy (RT) for prostate cancer (PCa) is associated with superior disease control, albeit limited by toxicity to organs at risk, including the rectum. For example, 5-year grade 2 or higher gastrointestinal (GI) toxicity in RTOG 0126 was increased by 40% in patients receiving 79.2 Gy versus 70 Gy (21% vs 15% for 79.2 vs 70.0 Gy, respectively; *P* < .01).^1^ Similarly, Kuban et al^2^ reported grade 2 or higher GI toxicity was doubled with dose escalation from 70 to 78 Gy.

Rectal toxicity has also been particularly notable in proton-based studies, with nearly 70% of patients reporting intermediate or poor bowel function with long-term follow-up in the proton radiation oncology group 95-09 trial.^3^, ^4^ Similarly, the University of Florida found that grade 2 or higher rectal bleeding occurred in more than 15% of patients treated with dose-escalated proton RT and that toxicity was correlated with rectal wall dose.^5^ One possible explanation for greater rectal toxicity seen in proton series is the sensitivity of proton radiation to daily setup variation from rectal filling, which may result in rectal wall doses that are higher than predicted on treatment planning software. When accounting for such uncertainties, prior reports have demonstrated that proton therapy results in greater high-dose (>50 Gy) rectal wall exposure compared to intensity-modulated radiation therapy (IMRT).^6^

Given the risk of rectal toxicity, various approaches at reducing toxicity have been explored, including rectal spacers (RS), which are associated with a decrease in bowel-related toxicity (13.8% vs 31.7% in photon-treated patients with vs without RS, respectively; *P* = .02).^7^ The utility of RS to mitigate rectal dose in proton patients was previously reported by Navaratnam et al^8^ where RS was associated with a 40% reduction in rectal volume receiving radiation. Additionally, post-treatment toxicity in proton patients was reduced from 19% with rectal balloon compared to 3% with rectal spacer, as reported by Dinh and colleagues.^9^ Among photon-treated patients, Mariados et al^10^ found that the rectal spacer increased the perirectal distance from 1.6 to 12.6 mm, which resulted in a nearly 10% reduction in rectal volume receiving 70 Gy and a decrease in late GI toxicity of 5%.^10^ These findings were independently replicated by Pieczonka et al, Hamstra et al, and Karsh et al.^11^, ^12^, ^13^

To date, no studies have quantified the relative benefit of RS in proton versus photon patients receiving dose-escalated RT. We hypothesized RS will confer a larger benefit in patients treated with proton therapy, compared with photon therapy, due to a greater benefit in mitigating rectal dose related to daily setup variation sensitivity and end-of-range relative biologic effectiveness unique to proton beam therapy.

## Methods

We conducted an Institutional review board-approved retrospective cohort analysis of patients with prostate adenocarcinoma receiving definitive volumetric arc therapy (VMAT)-based photon or pencil-beam scanning (PBS)-based proton external beam RT from 2018 to 2021 at a single institution.

We identified 4 patient cohorts: (1) photons with RS (Photon+RS), (2) photons without RS (Photon-RS), (3) protons with RS (Proton+RS), and (4) protons without RS (Proton-RS). Baseline demographic variables, including age, Gleason grade group, history of transurethral resection of the prostate, prostate gland volume, baseline urinary (eg, alpha-blocker, antispasmodic), and/or bowel (eg, antidiarrheal) modifying medication use, were assessed. Treatment-specific data, including the use of pelvic nodal irradiation and androgen deprivation therapy (ADT), were collected.

All patients received fractionated or moderately hypofractionated radiation with 60 Gy in 20 fractions, 70 Gy in 28 fractions, or 79.2 Gy in 44 fractions. When pelvic nodal irradiation was employed, it was given as a simultaneous integrated boost to 50.4 Gy when treating in 28 fractions and as an initial volume of 45 Gy in 25 fractions when utilizing 44 fractions. The clinical target volume for all patients included the prostate and proximal seminal vesicles. A separate clinical target volume, when needed, was created to encompass the pelvic lymph nodes. For photon patients, a planning target volume setup margin was employed for the prostate and proximal seminal vesicles that included a 7 to 8 mm isotropic expansion limited to 5 to 6 mm posteriorly, and the coverage goal for this planning target volume was for at least 95% of the volume to receive 100% of the prescription dose. For proton patients, an identical clinical target volume (CTV) was created, and a patient uncertainty margin of 5 mm isotropically was factored into treatment planning. Additionally, a 3.5% proton range uncertainty was added per facility protocol. The proton CTV coverage target was for at least 98% of the volume to receive ≥100% of the prescription dose. All proton doses were weighted with a fixed correction factor of 1.1 in accordance with International Committee on Radiation Units and Measurements 93 specifications. Daily imaging was at the discretion of the treating physician. For patients who did not have rectal spacer placement, most also did not have fiducial markers placed and were treated using daily cone-beam computed tomography (CT) scans for setup. In proton patients, quality assurance CT studies were obtained at least once during treatment to verify the daily treatment approximated the nominal treatment.

The primary endpoint was physician-reported and patient-reported toxicity. Toxicity assessments were completed at the following time points: baseline, <3 months post-treatment (ie, acute), ≥6 months post-treatment (ie, late), and at the most recent documented follow-up. Physician-reported toxicity was graded by common terminology criteria for adverse events (CTCAE) v5.0.^14^ Patient-reported toxicity was assessed via the International Prostate Symptom Score (IPSS)^15^ and Expanded Prostate Cancer Index Composite for Clinical Practice (EPIC-CP).^16^ Minimal clinically important difference (MCID) was defined as 0.5 standard deviations from baseline scores.

Clinicodemographic characteristics between the 4 cohorts were compared using analysis of variance or Kruskal-Wallis test for numerical variables and χ^2^ test or Fisher exact test for categorical variables. Gastrointestinal and genitourinary provider-reported toxicities were compared between RS and without RS groups among photon and proton cohorts separately by using χ^2^ test or Fisher exact test. Multivariable logistic regression was employed to estimate the odds ratio (OR) for GI toxicities for photon and proton cohorts. No multivariable analysis was performed for genitourinary toxicities as there were no significant differences between cohorts on univariable analysis. Mean and standard error of patient-reported toxicities were plotted as a function of time comparing RS and without RS cohorts. Multivariable linear mixed models were fitted to test whether there was any significant change over time or between comparing RS versus without RS cohorts. Tests were 2-sided with a 0.05 level of significance. All analyses were conducted using SAS version 9.4 (SAS Institute Inc, Cary, North Carolina).

## Results

### Demographics

We identified 164 patients eligible for analysis: 38 Photon-RS, 26 Proton-RS, 50 Photon+RS, and 50 Proton+RS. Median follow-up was 15.0 months, 21.1 months, 17.3 months, and 13.9 months for Photon+RS, Photon-RS, Proton+RS, and Proton-RS, respectively. All baseline clinicodemographic variables by treatment cohort are shown in Table 1.

**Table 1:** Descriptive statistics.

				Group				
Covariate	Statistics	Level	Photon+RS *N* = 50	Photon-RS *N* = 38	*P*-value^a^	Proton+RS *N* = 50	Proton-RS *N* = 26	*P*-value
Race	*N* (Col %)	White	26 (52.00)	4 (10.81)	**<.001**	27 (69.23)	11 (52.38)	.196
	*N* (Col %)	AA/other	24 (48.00)	33 (89.19)		12 (30.77)	10 (47.62)	
T stage	*N* (Col %)	T1	43 (86.00)	28 (73.68)	.370	34 (68.00)	15 (57.69)	**.061**
	*N* (Col %)	T2	5 (10.00)	6 (15.79)		14 (28.00)	5 (19.23)	
	*N* (Col %)	T3	2 (4.00)	3 (7.89)		2 (4.00)	5 (19.23)	
	*N* (Col %)	T4	0 (0)	1 (2.63)		0 (0)	1 (3.85)	
ADT	*N* (Col %)	No	25 (50.00)	7 (18.42)	**.002**	24 (48.00)	10 (38.46)	.428
	*N* (Col %)	Yes	25 (50.00)	31 (81.58)		26 (52.00)	16 (61.54)	
Risk group	*N* (Col %)	Low	1 (2.00)	0 (0)	**.045**	7 (14.00)	1 (3.85)	.244
	*N* (Col %)	Favorable intermediate	17 (34.00)	4 (10.53)		14 (28.00)	4 (15.38)	
	*N* (Col %)	Unfavorable intermediate	19 (38.00)	16 (42.11)		16 (32.00)	9 (34.62)	
	*N* (Col %)	High	11 (22.00)	15 (39.47)		7 (14.00)	8 (30.77)	
	*N* (Col %)	Very high	2 (4.00)	3 (7.89)		6 (12.00)	4 (15.38)	
PSA (cat.)	*N* (Col %)	<10	28 (56.00)	18 (47.37)	.489	39 (78.00)	16 (61.54)	**.036**
	*N* (Col %)	≥10-<20	15 (30.00)	11 (28.95)		10 (20.00)	5 (19.23)	
	*N* (Col %)	≥20	7 (14.00)	9 (23.68)		1 (2.00)	5 (19.23)	
Fields	*N* (Col %)	Prostate	43 (86.00)	25 (65.79)	**.025**	41 (82.00)	16 (61.54)	.051
	*N* (Col %)	Prostate+Pelvis	7 (14.00)	13 (34.21)		9 (18.00)	10 (38.46)	
Age	*N*		50	38	**.035**	50	26	.578
	Mean		70.56	67		68.82	69.85	
	Median		71	68		68.5	69	
	Min		55	56		47	50	
	Max		90	80		82	86	
	Std Dev		8.89	5.89		7.38	8.02	
PSA	*N*		50	38	.131	50	26	**.029**
	Mean		11.87	15.63		8.5	14.85	
	Median		9.62	10.20		7.65	6.53	
	Min		2.65	3.40		3.7	3.4	
	Max		56	58.80		35.60	93.10	
	Std Dev		9.03	14.05		5.10	18.95	

Notes: Baseline demographics for photon and proton-treated patients.

**Abbreviations:** Photon+RS, photon with rectoprostatic hydrogel; Photon-RS, photon without rectoprostatic hydrogel; Proton+RS, proton with rectoprostatic hydrogel; Proton-RS, proton without rectoprostatic hydrogel; ADT, androgen deprivation therapy; and PSA, prostate-specific antigen; AA, African American. P values <0.05 included in bold.

a The *P*-value is calculated by analysis of variance for numerical covariates and the χ2 test or Fisher exact test for categorical covariates.

There were significant differences between Photon+RS and Photon-RS cohorts in ADT usage (50.00% vs 81.58%, Photon+RS vs Photon-RS, respectively, *P* = .002), high or very high-risk group (26.00% vs 47.36%, Photon+RS vs Photon-RS, respectively, *P* = .045), pelvic nodal irradiation (14.00% vs 34.21%, Photon+RS vs Photon-RS, respectively, *P* = .025), and median age at treatment (71 years vs 68 years, Photon+RS vs Photon-RS, respectively, *P* = .035). The median (Q1-Q3) proportion of rectum receiving 65 Gy or higher (rectum V65Gy) was 1.80 Gy (0-6.00 Gy) for Photon+RS and 15.2 Gy (10.6 Gy-21.6 Gy) for Photon-RS patients (*P* < .001).

For patients treated with proton therapy, there were significant differences in baseline PSA >10.0 ng/mL (22.00% vs 38.46%, Proton+RS vs Proton-RS, respectively, *P* = .036), T stage (>T2 in 4.00% vs 23.08%, Proton+RS vs Proton-RS, respectively, *P* = .061) and a trend toward more frequent pelvic nodal treatment (18.00% vs 38.46%, Proton+RS vs Proton-RS, respectively, *P* = .051). Median (Q1-Q3) rectal V65Gy was 2.6 Gy (1.4 Gy-4.8 Gy) for Proton+RS patients and 3.2 (1.2-8 Gy) for Proton-RS patients (*P* = .314).

### Provider-reported toxicity

Acute CTCAE grade 1 to 2 GI toxicity was significantly higher in the Proton-RS cohort compared with Proton+RS: 30.77% versus 6.12%, respectively (*P* = .009). There was numerically higher late grade 1 to 2 GI toxicity with Proton-RS compared to Proton+RS, although this did not reach statistical significance (26.09% vs 8.51%, respectively [*P* = .08]) (Table 2). Additionally, most recent grade 1 to 2 GI toxicity remained higher in the Proton-RS cohort (26.09% and 4.26% in Proton-RS vs Proton+RS, respectively; *P* = .01). There was no significant difference between Proton-RS versus Proton+RS cohorts in acute, late, or most recent genitourinary toxicity (Table 2). On sensitivity analysis stratified by treatment of pelvic lymph nodes, CTCAE GI toxicity in patients receiving treatment to the prostate only remained higher in Proton-RS versus Proton+RS patients at acute (2.4% vs 37.6%, *P* = .002) and most recent (3% vs 50%, *P* = .001) time points.

**Table 2:** Provider-reported toxicity—proton.

			Group		
Covariate	Statistics	Level	Proton+RS *N* = 50	Proton-RS *N* = 26	*P*-value^a^
A-GU	*N* (Col %)	0	6 (12.24)	1 (3.85)	.59
	*N* (Col %)	1	10 (20.41)	6 (23.08)	
	*N* (Col %)	2+	33 (67.35)	19 (73.08)	
A-GI	*N* (Col %)	0	46 (93.88)	18 (69.23)	**.01**
	*N* (Col %)	1	3 (6.12)	6 (23.08)	
	*N* (Col %)	2+	0 (0)	2 (7.69)	
L-GU	*N* (Col %)	0	22 (46.81)	6 (26.09)	.15
	*N* (Col %)	1	4 (8.51)	1 (4.35)	
	*N* (Col %)	2+	21 (44.68)	16 (69.57)	
L-GI	*N* (Col %)	0	43 (91.49)	17 (73.91)	.08
	*N* (Col %)	1	4 (8.51)	5 (21.74)	
	*N* (Col %)	2+	0 (0)	1 (4.35)	
R-GU	*N* (Col %)	0	25 (53.19)	6 (26.09)	.08
	*N* (Col %)	1	2 (4.26)	1 (4.35)	
	*N* (Col %)	2+	20 (42.55)	16 (69.57)	
R-GI	*N* (Col %)	0	45 (95.74)	17 (73.91)	**.01**
	*N* (Col %)	1	2 (4.26)	5 (21.74)	
	*N* (Col %)	2+	0 (0)	1 (4.35)	

Notes: Univariable analysis of CTCAE v5.0 toxicity at specified time points in proton-treated patients.

**Abbreviations:** Proton+RS, proton with rectal spacer; Proton-RS, proton without rectal spacer; and A/L/R GU and GI, acute (<3 months), max late (≥3 months), most recent (≥3 months) genitourinary and gastrointestinal toxicities, respectively. P values <0.05 included in bold.

a The *P*-value is calculated by χ2 test or Fisher exact, where appropriate.

There were no significant differences between Photon-RS versus Photon+RS cohorts in acute, late, or most recent grade 1 to 2 GI or genitourinary toxicity (Table 3). On sensitivity analysis stratified by treatment of pelvic lymph nodes, there remained no significant difference between Photon+RS versus Photon-RS cohorts in grade 1 to 2 CTCAE GI or genitourinary toxicity at any time point.

**Table 3:** Provider-reported toxicity—photon.

			Group		
Covariate	Statistics	Level	Photon+RS *N* = 50	Photon-RS *N* = 38	*P*-value^a^
A-GU	*N* (Col %)	0	6 (12.50)	5 (13.51)	.90
	*N* (Col %)	1	11 (22.92)	7 (18.92)	
	*N* (Col %)	2+	31 (64.58)	25 (67.57)	
A-GI	*N* (Col %)	0	33 (68.75)	26 (70.27)	1.000
	*N* (Col %)	1	13 (27.08)	10 (27.03)	
	*N* (Col %)	2+	2 (4.17)	1 (2.70)	
L-GU	*N* (Col %)	0	13 (28.89)	8 (21.05)	.27
	*N* (Col %)	1	4 (8.89)	9 (23.68)	
	*N* (Col %)	2+	28 (62.22)	21 (55.26)	
L-GI	*N* (Col %)	0	35 (77.78)	32 (84.21)	.06
	*N* (Col %)	1	9 (20.00)	2 (5.26)	
	*N* (Col %)	2+	1 (2.22)	4 (10.53)	
R-GU	*N* (Col %)	0	20 (44.44)	10 (26.32)	.27
	*N* (Col %)	1	4 (8.89)	7 (18.42)	
	*N* (Col %)	2+	21 (46.66)	21 (55.26)	
R-GI	*N* (Col %)	0	38 (84.44)	35 (92.11)	.56
	*N* (Col %)	1	6 (13.33)	2 (5.26)	
	*N* (Col %)	2+	1 (2.22)	1 (2.63)	

Notes: Univariable analysis of CTCAE v5.0 toxicity at specified time points in photon-treated patients.

**Abbreviations:** Photon+RS, proton with rectal spacer; Photon-RS, photon without rectal spacer; and A/L/R GU and GI, acute (<3 months), max late (≥3 months), most recent (≥3 months) genitourinary and gastrointestinal toxicities, respectively.

a The *P*-value is calculated by the χ2 test or Fisher exact, where appropriate.

#### Multivariable analysis

Among patients treated with proton therapy, RS use was associated with significantly lower odds of acute, late, and most recent grade ≥1 GI toxicity (Proton+RS vs Proton-RS: acute adjusted OR 0.10 [*P* < .01], late adjusted OR 0.23 [*P* = .05], and most recent adjusted OR 0.11 [*P* = .02]; Supplementary Table 1). Urinary/bowel medication use, radiation treatment field (prostate only vs whole pelvis), prostate CTV volume, prostate volume, ADT use, and age were not associated with acute, late, or most recent GI toxicity in proton patients.

In patients treated with VMAT, RS use was not significantly associated with acute, late, or most recent GI toxicity. Prostate-only, compared with the whole pelvis, treatment was associated with higher odds of grade ≥1 acute GI toxicity (OR 0.26, *P* = .04; Supplementary Table 2); however, this difference did not persist at late or most recent follow-up, and only 16 photon patients received pelvic nodal treatment. Urinary/bowel medication use, prostate volume, ADT use, and age were not associated with acute, late, or most recent GI toxicity.

### Patient-reported toxicity

#### International Prostate Symptom Score

**Figure 1:**
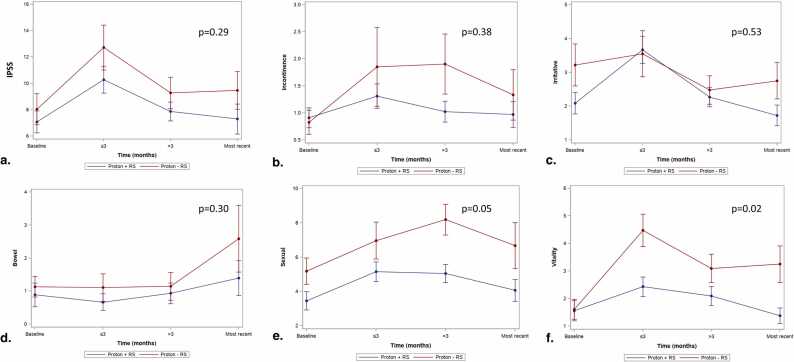
Linear mixed model plots with mean (standard error) for proton patients with or without rectoprostatic hydrogel spacer. (a) International Prostate Symptom Score, (b) Expanded Prostate Cancer Index Composite for Clinical Practice (EPIC-CP) incontinence, (c) EPIC-CP irritative, (d) EPIC-CP bowel, (e) EPIC-CP sexual, and (f) EPIC-CP vitality scores. Abbreviations: IPSS, International Prostate Symptom Score; RS, rectal spacers.

**Figure 2:**
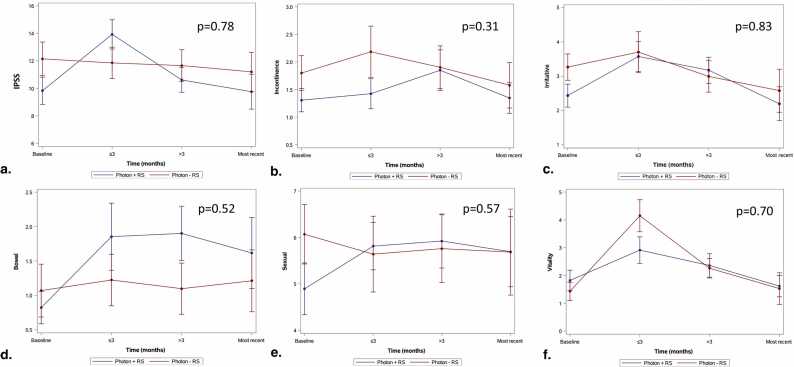
Linear mixed model plots with mean (standard error) for photon patients with or without rectoprostatic hydrogel spacer. (a) International Prostate Symptom Score, (b) Expanded Prostate Cancer Index Composite for Clinical Practice (EPIC-CP) incontinence, (c) EPIC-CP irritative, (d) EPIC-CP bowel, (e) EPIC-CP sexual, and (f) EPIC-CP vitality scores. Abbreviations: IPSS, International Prostate Symptom Score; RS, rectal spacers.

There were no significant differences in IPSS scores between Proton-RS versus Proton+RS or Photon-RS versus Photon+RS patients (Figures 1a and 2a, respectively). Baseline urinary-modifying medication use was associated with worse IPSS scores over time (+3.70 [*P* < .01] and +2.67 [*P* = .02] for photon and proton patients, respectively) with photon patient scores surpassing the MCID threshold (Supplementary Tables 3 and 4 for proton and photon patients, respectively).

#### Expanded Prostate Cancer Index Composite for Clinical Practice incontinence

There were no significant differences in EPIC-CP incontinence scores between Proton-RS versus Proton+RS or Photon-RS versus Photon+RS patients (Figures 1b and 2b, respectively). Baseline urinary-modifying medication use was associated with higher incontinence scores in photon patients (+0.99, *P* = .01) with the difference exceeding the MCID threshold; however, no other covariables were significantly associated with EPIC-CP incontinence scores.

#### Expanded Prostate Cancer Index Composite for Clinical Practice irritative

There were no significant differences in EPIC-CP irritative scores between Proton-RS versus Proton+RS or Photon-RS versus Photon+RS patients (Figures 1c and 2c, respectively). Baseline urinary-modifying medication use was associated with higher (ie, worse) irritative scores for photon (+1.73, *P* < .01) and proton (+0.96, *P* = .02) patients; however, only the difference in photon patients exceeded the MCID threshold (Supplementary Tables 3 and 4 for proton and photon patients, respectively).

#### Expanded Prostate Cancer Index Composite for Clinical Practice bowel

There were no significant differences in EPIC-CP bowel scores between Proton-RS versus Proton+RS or Photon-RS versus Photon+RS patients (Figures 1d and 2d, respectively). Baseline bowel-modifying medication use was associated with higher (ie, worse) bowel bother scores in photon patients (0.84, *P* = .05); however, the difference did not exceed the MCID threshold. Use of RS was not associated with differences in either photon (*P* = .52) or proton (*P* = .30) patients.

#### Expanded Prostate Cancer Index Composite for Clinical Practice sexual

There were no significant differences in EPIC-CP sexual scores between Proton-RS versus Proton+RS or Photon-RS versus Photon+RS patients (Figures 1e and 2e, respectively). Use of RS was associated with lower sexual scores in proton patients (−1.48, *P* = .05; Supplementary Table 3); however, this difference did exceed the MCID. Additionally, baseline urinary-modifying medication use was associated with higher (ie, worse) sexual bother scores in proton patients (+1.81, *P* = .02) with the difference approaching the MCID of 1.84. Age was also associated with higher (ie, worse) sexual bother scores in proton patients (+0.09, *P* = .04); however, the difference did not exceed the MCID threshold.

#### Expanded Prostate Cancer Index Composite for Clinical Practice vitality

Use of RS was associated with lower (ie, better) vitality scores in proton patients (−0.98, *P* = .02), though the difference did not exceed the MCID (Supplementary Table 3; Figure 1f). Use of ADT was associated with higher (ie, worse) vitality scores in proton patients (+1.14, *P* = .01) with the difference exceeding the MCID. Use of urinary-modifying medications at baseline was associated with higher (ie, worse) vitality scores in photon patients (+0.95, *P* = .04); however, this difference did not exceed the MCID threshold.

## Discussion

In this retrospective institutional analysis of PCa men treated with definitive fractionated external beam RT with either protons or photons, rectal spacer use was associated with a lower provider-reported acute, late, and most recent CTCAE grade 1+ GI toxicity in Proton+RS compared to Proton-RS patients. In contrast, there was no significant difference in GI toxicity in photon patients with versus without RS. Prospective validation in larger cohorts is needed, but these findings support the hypothesis that, for men with PCa undergoing moderately hypofractionated external beam radiation, rectal spacers may have a relatively greater impact in reducing bowel toxicity with proton therapy compared with photon therapy (ie, VMAT).

Multiple prior reports have suggested higher GI toxicity in proton patients compared to IMRT patients. For example, Pan et al^17^ conducted a propensity-matched comparative analysis between men with PCa treated with proton versus IMRT using the MarketScan Commercial Claims and Encounters (IBM, Armonk, New York) database and found an increased risk of bowel toxicity at 2 years with proton therapy compared with IMRT (15.3% vs 9.7%; *P* < .001), which was primarily related to rectal bleeding. Additionally, Sheets, Goldin et al^18^ reported a one-third lower GI toxicity with IMRT compared to protons, though this work was based on surveillance, epidemiology, and end-results Medicare-linked data, which do not include treatment-related variables such as prescribed radiation dose, use of androgen deprivation, or use of brachytherapy. Conversely, reports by Yu et al^19^ and Fang et al^20^ demonstrated similar GI toxicity among patients receiving proton versus photon therapy. Overall, there remains substantial uncertainty about the relative risk of toxicity between photon and proton treatments. It is important to note that these studies included men treated in an era of passive scatter protons, which have less capability of dose modulation around the anterior rectal wall compared to PBS.^21^, ^22^ Our study utilized a contemporary cohort of men treated after 2018 exclusively with PBS proton therapy.

Gastrointestinal toxicity following proton therapy may be a result of multiple factors. As demonstrated by Zhang et al,^6^ nominal proton plans may expose a higher volume of the rectum to doses ≥50 Gy and are extremely sensitive to variations in daily setup. Additionally, as proton plans are robustly optimized for target coverage to account for setup uncertainty and resultant variations in tissue density along the beam path, there may be a higher dose exposure to the rectum than is displayed on nominal plans. This may partially explain the difference in rectal dose reduction observed between photon and proton cohorts. Overall, compared to photon beams modulated with multileaf collimators, proton beams tend to have a larger penumbra, which may increase the volume of normal tissue exposed to high-dose radiation.^23^ Additionally, due to the prolonged treatment time with proton therapy, influenced by beam-sharing across multiple rooms), intrafraction motion may also contribute to unrecognized setup uncertainties. Taken together, this array of factors may all contribute to an increased risk of bowel toxicity with proton therapy.

Several important limitations of our study should be considered. First, the use of RS was heterogeneous among patients. Specifically, patients who received RS tended to be lower risk and thus less likely to receive pelvic nodal treatment or ADT. Additionally, larger proportions of non-White patients were treated without RS in both photon and proton cohorts, possibly due to higher risk disease presentation in these patients with risk for extracapsular extension that precluded the safety of RS implantation.^24^, ^25^ These differences reflect provider preference and are likely influenced by the exclusion of high-risk patients in initial prospective studies of RS that led to FDA approval.^10^ The use of pelvic nodal irradiation was unbalanced with a higher proportion of patients without rectal spacer receiving pelvic nodal treatment in both the photon (14% vs 34%, Photon+RS and Photon-RS, respectively) and proton (18% vs 38%, Proton+RS and Proton-RS, respectively); however, the proportion of pelvic nodal radiation use was consistent in both populations. As such, we would anticipate the increased risk of GI toxicity related to larger treatment fields to be similar in both photon and proton patients, which was not seen. In addition, whole pelvic treatment was not associated with increased GI toxicity among proton patients on MVA. One potential explanation for the observed difference in toxicity between whole pelvic and prostate-only fields is the use of daily image guidance, which was not assessed in this report. Most patients who did not have RS placement also did not have fiducial marker placement and thus had daily Cone-beam CT for setup verification. Utilization of daily Cone-beam CT may have advantages that could help mitigate rectal toxicity, such as daily assessment of rectal filling. Finally, follow-up duration was limited across patient cohorts with median follow-up ranging from approximately 1.5 to 2 years, which may not capture the full extent of late treatment-related toxicity. Specifically, Kim et al^26^ reported the incidence of GI toxicity following prostate RT to continue rising in a near linear fashion beyond 2 years. However, multiple reports have utilized 24-month assessments to evaluate late toxicity, and “most-recent” toxicity data were included to provide the most matured data in this contemporary data set.^27^, ^28^, ^29^

In conclusion, our analysis demonstrates a relatively greater impact of RS use in mitigating rectal toxicity in PCa men treated with PBS proton therapy compared with VMAT photon therapy. These findings support the benefit of RS use in mitigating bowel toxicity among PCa patients treated with dose-escalated proton radiation. While we did not find a significant difference in GI toxicity in patients treated with photon therapy, overall rates of toxicity were low, which limits study power.

## Author Contributions

Vishal R. Dhere: Conception, Methodology, Data curation, Writing- Original draft, Writing- Review and Editing. Subir Goyal: Data curation, Formal analysis, Visualization, Writing- Original draft, Writing- Review and Editing. Jun Zhou, Nikhil T. Sebastian, Ashish B. Patel, Sheela Hanasoge, Pretesh R. Patel, Joseph Shelton, Karen D. Godette, and Bruce W. Hershatter: Writing- Review and Editing. Ashesh B. Jani: Conception, Writing- Review and Editing. Sagar A. Patel: Conception, Data curation, Writing- Original draft, Writing - Review and Editing.

## Declaration of Conflicts of Interest

The authors declare the following financial interests/personal relationships which may be considered as potential competing interests: Subir Goyal reports financial support was provided by the National Institutes of Health. If there are other authors, they declare that they have no known competing financial interests or personal relationships that could have appeared to influence the work reported in this paper.

## Data Availability

Research data are stored in an institutional repository and will be shared upon request to the corresponding author.

## Footnotes

Supplementary material associated with this article can be found in the online version at doi:10.1016/j.ijpt.2024.100111.
