# Supplementary material for: Impact of Rectal Spacer on Toxicity Reduction in Men Treated With Proton Versus Photon Therapy
Source: Int J Part Ther. 2024 Jun 20;13:100111. doi: 10.1016/j.ijpt.2024.100111 (PMC11283227; doi:10.1016/j.ijpt.2024.100111)
Supplement: Supplementary file 1 — Supplementary material [file mmc1.pdf]

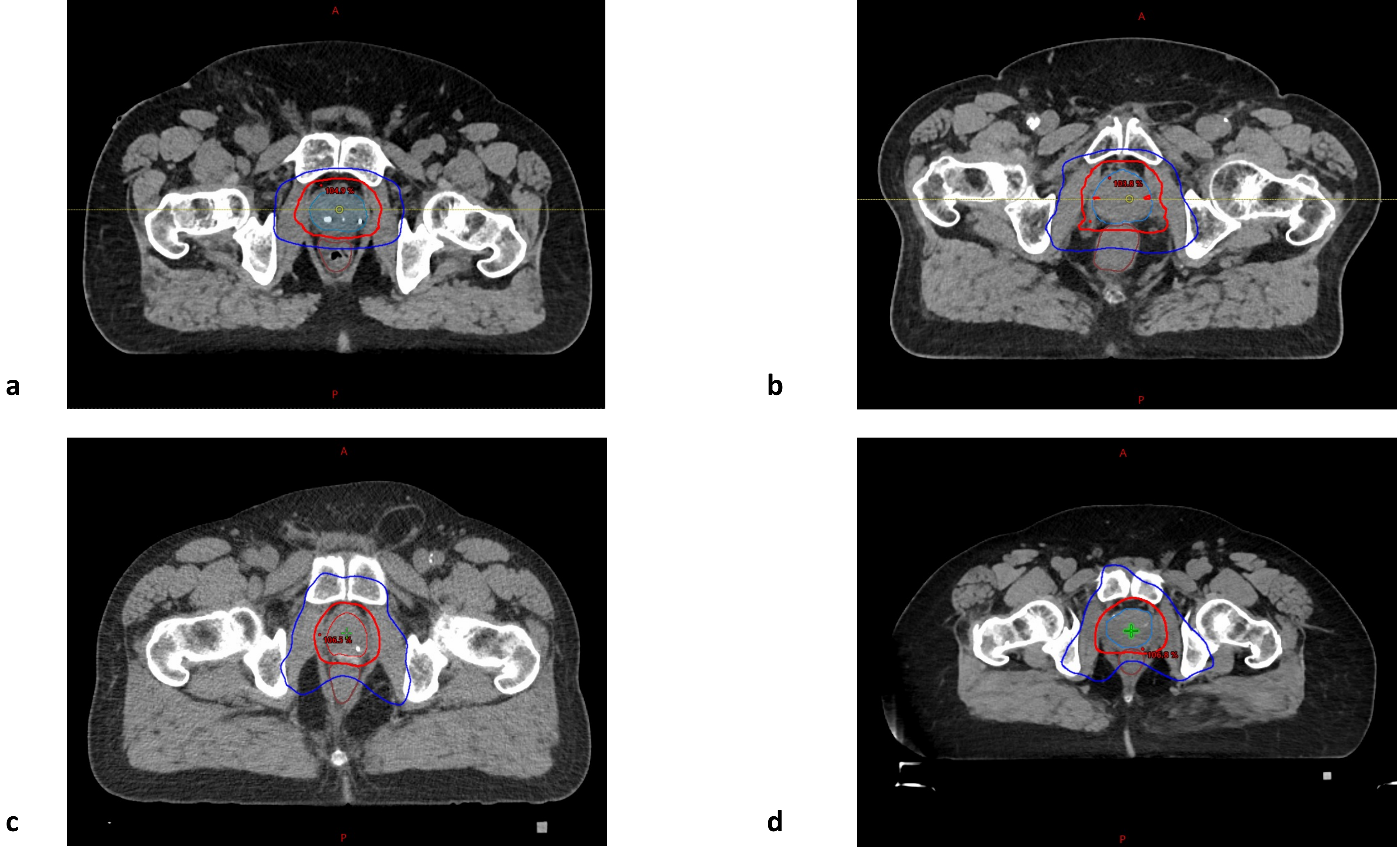

Supplemental figure 1: Representative axial images including isodose distributions for a. Proton+RS, b. Proton-RS, c. Photon+RS and d. Photon-RS. Prostate CTV (light blue) and rectal (brown) contours are included. Isodoses- Red: 100%; Blue: 50%
