# Supplementary material for: Impact of Rectal Spacer on Toxicity Reduction in Men Treated With Proton Versus Photon Therapy
Source: Int J Part Ther. 2024 Jun 20;13:100111. doi: 10.1016/j.ijpt.2024.100111 (PMC11283227; doi:10.1016/j.ijpt.2024.100111)
Supplement: Supplementary file 2 — Supplementary material [file mmc2.docx]

|  | Supplemental Table 1- Multivariable Logistic Regression for Provider Reported CTCAE V5.0 Gastrointestinal Toxicity - Proton | | | | | | | | | | |  |
| --- | --- | --- | --- | --- | --- | --- | --- | --- | --- | --- | --- | --- |
|  | | | Acute GI | | | Late GI | | | Most Recent GI | | | |
|  |  |  | N | Odds Ratio (95% CI) | OR P-value | N | Odds Ratio (95% CI) | OR P-Value | N | Odds Ratio (95% CI) | OR P-value | |
| Arm | | Proton+RS | 48 | 0.10 (0.02-0.45) | **<0.01** | 47 | 0.23 (0.05-1.02) | **0.05** | 47 | 0.11 (0.02-0.66) | **0.02** | |
|  |  | Proton-RS | 26 |  |  | 23 |  |  | 23 |  |  | |
| Baseline medication use | | Yes | 25 | 0.80 (0.16-3.89) | 0.78 | 24 | 0.52 (0.12-2.26) | 0.38 | 24 | 0.66 (0.12-3.49) | 0.62 | |
|  |  | No | 49 |  |  | 46 |  |  | 46 |  |  | |
| Fields | | Whole pelvis | 17 | 0.98 (0.16-6.05) | 0.98 | 16 | 2.26 (0.31-16.42) | 0.42 | 16 | 1.79 (0.22-14.42) | 0.58 | |
|  |  | Prostate only | 57 |  |  | 54 |  |  | 54 |  |  | |
| Androgen Deprivation  (Yes vs **No**) | | With ADT | 40 | 1.87 (0.39-8.93) | 0.43 | 37 | 1.23 (0.28-5.53) | 0.78 | 37 | 1.47 (0.27-8.14) | 0.66 | |
|  |  | Without ADT | 34 |  |  | 33 |  |  | 33 |  |  | |
| Prostate CTV volume  (Continuous) | |  | 74 | 0.99 (0.96-1.02) | 0.51 | 70 | 1.00 (0.97-1.03) | 0.87 | 70 | 1.01 (0.97-1.04) | 0.78 | |
| Age  (Continuous) | |  | 74 | 1.05 (0.95-1.15) | 0.36 | 70 | 1.02 (0.92-1.13) | 0.73 | 70 | 1.02 (0.90-1.15) | 0.77 | |

Supplemental Table 1- Multivariable analysis of provider reported CTCAE v5.0 gastrointestinal (GI) toxicity at specified time points in proton treated patients. Proton+RS: Proton with rectoprostatic hydrogel, Proton-RS: Proton without rectoprostatic hydrogel.
