# Supplementary material for: Impact of Rectal Spacer on Toxicity Reduction in Men Treated With Proton Versus Photon Therapy
Source: Int J Part Ther. 2024 Jun 20;13:100111. doi: 10.1016/j.ijpt.2024.100111 (PMC11283227; doi:10.1016/j.ijpt.2024.100111)
Supplement: Supplementary file 3 — Supplementary material [file mmc3.docx]

| Supplemental Table 2- Multivariable Logistic Regression for Provider Reported CTCAE V5.0 Toxicity - Photon | | | | | | | |  | |  | |  | |  |
| --- | --- | --- | --- | --- | --- | --- | --- | --- | --- | --- | --- | --- | --- | --- |
|  | | Acute GI | | | Late GI | | | | Most Recent GI | | | | | |
|  |  | N | Odds Ratio (95% CI) | OR P-value | N | Odds Ratio (95% CI) | OR P-Value | | N | | Odds Ratio (95% CI) | | OR P-value | |
| Arm | Photon+RS | 45 | 0.72 (0.24-2.17) | 0.56 | 43 | 0.90 (0.26-3.12) | 0.87 | | 43 | | 0.69 (0.14-3.31) | | 0.64 | |
|  | Photon-RS | 37 |  |  | 37 |  |  | | 37 | |  | |  | |
| Baseline medication use | Yes | 24 | 0.51 (0.18-1.48) | 0.22 | 24 | 0.87 (0.25-3.00) | 0.82 | | 24 | | 1.54 (0.28-8.56) | | 0.62 | |
|  | No | 58 |  |  | 56 |  |  | | 56 | |  | |  | |
| Fields | Whole pelvis | 18 | 0.26 (0.07-0.97) | 0.04 | 16 | 3.77 (0.42-33.91) | 0.24 | | 16 | | 1.79 (0.22-14.42) | | 0.58 | |
|  | Prostate only | 64 |  |  | 64 |  |  | | 64 | |  | |  | |
| Androgen Deprivation  (Yes vs **No**) | With ADT | 53 | 1.55 (0.45-5.37) | 0.50 | 53 | 1.17 (0.34-4.07) | 0.81 | | 53 | | 1.21 (0.28-5.33) | | 0.80 | |
|  | Without ADT | 29 |  |  | 27 |  |  | | 27 | |  | |  | |
| Prostate CTV volume  (Continuous) |  | 82 | 1.00 (0.98-1.02) | 0.75 | 80 | 1.00 (0.97-1.02) | 0.94 | | 80 | | 1.02 (0.98-1.06) | | 0.29 | |
| Age  (Continuous) |  | 82 | 1.06 (0.98-1.14) | 0.13 | 80 | 1.01 (0.93-1.09) | 0.89 | | 80 | | 1.00 (0.91-1.10) | | 0.97 | |

Supplemental Table 2- Multivariable analysis of provider reported CTCAE v5.0 gastrointestinal (GI) toxicity at specified time points. Photon+RS: Photon with rectoprostatic hydrogel, Photon-RS: Photon without rectoprostatic hydrogel.
