# Supplementary material for: Impact of Rectal Spacer on Toxicity Reduction in Men Treated With Proton Versus Photon Therapy
Source: Int J Part Ther. 2024 Jun 20;13:100111. doi: 10.1016/j.ijpt.2024.100111 (PMC11283227; doi:10.1016/j.ijpt.2024.100111)
Supplement: Supplementary file 4 — Supplementary material [file mmc4.docx]

|  | IPSS | EPIC-CP  Incontinence | EPIC-CP  Irritative | EPIC-CP  Bowel | EPIC-CP  Sexual | EPIC-CP  Vitality |
| --- | --- | --- | --- | --- | --- | --- |
| MCID | 2.86 | 0.59 | 1.25 | 1.06 | 1.84 | 1.11 |
| Arm  (With vs. **Without RS**) | -1.24 (p=0.29) | -0.48  (p=0.38) | -0.27  (p=0.53) | -0.47  (p=0.30) | **-1.48**  **(p=0.05)** | **-0.98**  **(p=0.02)** |
| Baseline GI/GU medication use  (Yes vs **No)** | **2.67**  **(p=0.02)** | 0.26  (p=0.51) | **0.96**  **(p=0.02)** | 0.28  (p=0.54) | **1.81**  **(p=0.02)** | 0.38  (p=0.36) |
| Fields  (Whole pelvic vs **Prostate only**) | -1.62  (p=0.24) | 0.01  (p=0.98) | -0.30  (p=0.56) | -0.45  (p=0.41) | 1.08  (p=0.24) | 0.02  (p=0.97) |
| Androgen Deprivation  (Yes vs **No**) | 1.91  (p=0.09) | -0.05  (p=0.89) | 0.46  (p=0.27) | 0.17  (p=0.70) | <0.01  (p=1.00) | **1.14**  **(p=0.01)** |
| Prostate CTV volume  (Continuous) | 0.04  (p=0.07) | 0.01  (p=0.29) | 0.01  (p=0.47) | <0.01  (p=0.44) | 0.01  (p=0.37) | <0.01  (p=0.97) |
| Age  (Continuous) | 0.06  (p=0.42) | -0.01  (p=0.75) | -0.02  (p=0.56) | <0.01  (p=0.88) | **0.09**  **(p=0.04)** | -0.03  (p=0.18) |

Supplemental Table 3- Multivariable Linear Mixed Effects Models for Patient Reported Toxicity - Proton

Supplemental Table 3: Multivariable analysis of proton treated patients including specified covariables. Bolded covariates were used as baseline for comparisons. Baseline GU medications were used for IPSS, EPIC-CP incontinence, irritative, sexual and vitality scores while baseline GI medications were used for EPIC-CP bowel scores. Values represent absolute difference in score for each category. IPSS: International Prostate Symptom Score; EPIC-CP: Expanded Prostate Cancer Index for Clinical Practice; MCID: Minimal clinically important difference.
