# Supplementary material for: Impact of Rectal Spacer on Toxicity Reduction in Men Treated With Proton Versus Photon Therapy
Source: Int J Part Ther. 2024 Jun 20;13:100111. doi: 10.1016/j.ijpt.2024.100111 (PMC11283227; doi:10.1016/j.ijpt.2024.100111)
Supplement: Supplementary file 5 — Supplementary material [file mmc5.docx]

|  | IPSS | EPIC-CP  Incontinence | EPIC-CP  Irritative | EPIC-CP  Bowel | EPIC-CP  Sexual | EPIC-CP  Vitality |
| --- | --- | --- | --- | --- | --- | --- |
| MCID | 3.50 | 0.76 | 1.07 | 0.87 | 1.72 | 1.04 |
| Arm  (With vs. **Without RS**) | -0.34  (p=0.78) | -0.39  (p=0.31) | -0.09  (p=0.83) | 0.27  (p=0.52) | -0.44  (p=0.57) | -0.18  (p=0.70) |
| Baseline GI/GU medication use  (Yes vs **No)** | **3.70**  **(p<0.01)** | **0.99**  **(p=0.01)** | **1.73**  **(p<0.01)** | **0.84**  **(p=0.05)** | 0.80  (p=0.32) | **0.95**  **(p=0.04)** |
| Fields  (Whole pelvic vs **Prostate only**) | 1.04  (p=0.49) | 0.10  (p=0.84) | 0.43  (p=0.44) | -0.45  (p=0.41) | 0.34  (p=0.73) | 0.56  (p=0.33) |
| Androgen Deprivation  (Yes vs **No**) | 0.02  (p=0.99) | -0.20  (p=0.863) | -0.02  (p=0.97) | 0.22  (p=0.63) | -0.02  (p=0.98) | 0.52  (p=0.30) |
| Prostate CTV volume  (Continuous) | 0.02  (p=0.42) | <0.01  (p=0.51) | 0.01  (p=0.48) | <0.01  (p=0.54) | -0.01  (p=0.70) | -0.01  (p=0.10) |
| Age  (Continuous) | -0.04  (p=0.65) | 0.03  (p=0.18) | -0.01  (p=0.83) | <0.01  (p=1.00) | 0.07  (p=0.20) | -0.01  (p=0.64) |

Supplemental Table 4- Multivariable Linear Mixed Effects Models for Patient Reported Toxicity - Photon

Supplemental table 4: Multivariable analysis for photon treated patients including specified covariables. Bolded covariates were used as baseline for comparisons. Baseline GU medications were used for IPSS, EPIC-CP incontinence, irritative, sexual and vitality scores while baseline GI medications were used for EPIC-CP bowel scores. Values represent absolute difference in score for each category. IPSS: International Prostate Symptom Score; EPIC-CP: Expanded Prostate Cancer Index for Clinical Practice; MCID: Minimal clinically important difference, based on distributive approach of 0.5 standard deviation at baseline.
